# Evaluation of Genetic Susceptibility Loci for Chronic Hepatitis B in Chinese: Two Independent Case-Control Studies

**DOI:** 10.1371/journal.pone.0017608

**Published:** 2011-03-08

**Authors:** Li Wang, Xiao-Pan Wu, Wei Zhang, Da-Hai Zhu, Ying Wang, Yan-Ping Li, Yao Tian, Rong-Cheng Li, Zhuo Li, Xinlin Zhu, Jun-Hong Li, Jun Cai, Li Liu, Xiao-Ping Miao, Ying Liu, Hui Li

**Affiliations:** 1 Department of Epidemiology, Institute of Basic Medical Sciences, Chinese Academy of Medical Sciences, School of Basic Medicine, Peking Union Medical College, Beijing, China; 2 National Laboratory of Medical Molecular Biology, Institute of Basic Medical Sciences, Chinese Academy of Medical Sciences, School of Basic Medicine, Peking Union Medical College, Beijing, China; 3 Beijing Center for Disease Control and Prevention, Beijing, China; 4 Department of Epidemiology and Biostatistics and Ministry of Education Key Lab of Environment and Health, School of Public Health, Tongji Medical College, Huazhong University of Science and Technology, Wuhan, Hubei, China; 5 Guangxi Center for Disease Control and Prevention, Nanning, Guangxi, China; 6 Department of Infectious Disease, Capital University of Medical Science, Affiliated Youan Hospital, Beijing, China; 7 Chinese Academy of Science Key Laboratory of Genome Sciences and Information, Beijing Institute of Genomics, Chinese Academy of Sciences, Beijing, China; 8 Department of Virology, Wuhan Centers for Disease Prevention and Control, Wuhan, Hubei, China; Singapore Institute for Clinical Sciences, Singapore

## Abstract

**Background:**

A recent genome-wide scan has identified two genetic variants in the HLA-DP region strongly associated with hepatitis B infection in Japanese. This study evaluates the effects of these risk variants in Chinese, where the HBV infection is the most popular in the world.

**Methods and Findings:**

We have assessed the relationship between these two single nucleotide polymorphisms (rs3077 and rs9277535) and chronic hepatitis B infection in two independent case-control studies. The first population in Chinese Han included 736 patients and 782 spontaneously recovered controls. The second set was established in Chinese Zhuang minority of 177 patients and 208 controls. Both A alleles of rs3077 and rs9277535 significantly deceased the risk to CHB in Chinese Han (OR = 0.540, 95%CI: 0.464–0.628, *P* = 4.068×10^−16^ and OR = 0.696, 95%CI: 0.601–0.806, *P* = 1.062×10^−6^, respectively). Conceivably, rs9277535 was found to be associated with decreased risk of the disease in Chinese Zhuang, with an OR of 0.606 (95%CI, 0.441–0.833, *P* = 0.002).

**Conclusion:**

Chronic hepatitis B susceptibility loci in HLA-DP region (rs3077 and rs9277535) identified by genome-wide scan in Japanese population were validated in Chinese population. These findings might provide clues to develop screening and surveillance strategies.

## Introduction

Hepatitis B virus (HBV) infection is the most common cause of liver disease, with around 2 billion people infected and 350 million suffering from chronic HBV infection worldwide [1]. Its prevalence shows a regional diversity, with relative low-incidence in Western countries but endemic in China [2], where a national survey reported roughly 7.18% rate of HBsAg carriers in the general population, indicating more than one-third of the world's HBV carriers live in China [3]. The clinical outcome of HBV infection is also variable, from spontaneously recovery to persistent infection that may increasingly progress to cirrhosis and hepatocellular carcinoma. It is estimated that more than 300,000 people die from HBV-related diseases in China annually, including 180,000 patients with hepatocellular carcinoma, which is associated with increased healthcare challenge and other socioeconomic burdens. The mechanisms underlying the different clinical outcomes of HBV infection have not been fully understood. Although environmental factors such as viral strain, gender, infection age and immune status of the host were suspected to affect risk of chronic hepatitis B (CHB) [4], there were evidence strongly suggested that host genetic factors [5]–[7] may play an important role in the occurrence of CHB. Less than 20% people exposed to HBV develop to CHB, and only a fraction of exposed people actually develops serious clinical sequelae, such as cirrhosis and and hepatocellular carcinoma, during their lifespan, indicating that genetic susceptibility factors may influence development of CHB [6] and a strong selection in genetic evolution limit its prevalence. Additional, familial studies indicated that monozygotic twins, dizygotic twins and general control may suffer from infection outcome differently and female sibling may have more chance to develop natural immunity and recover easily [5].

During the past decades, researchers have paid much attention to genetic polymorphisms (SNPs) in both human leukocyte antigen (HLA) genes and some other genes which modulate or control immune response to HBV infection. For example, accumulating evidence, including our former studies, has demonstrated that SNPs in tumor necrosis factor-alpha [8]–[13], vitamin D receptor gene [13]–[15] and HLA-DR1 [11], [16]–[20] may be associated with the outcomes of HBV infections. However, most of the results from previous studies so far remain inconsistent due to disparities in ethnicity, sample size and genotyping techniques.

Most recently, a two-stage genome-wide association (GWAS) study using 786 Japanese CHB and 2201 controls was performed by Kamatani Y et al., then the identified SNPs, including rs3077 and rs9277535 located in HLA-DPA1 and DPB1, were validated in three additional Japanese and Thai cohorts consisting 1300 patients and 2100 controls [21]. HLA-DPA1 and HLA-DPB1 encode the HLA-DP alpha and beta chains and may be implicated in antigen presentation to CD4^+^ positive T lymphocyte, which is important for HBV clearance, whiles the clear mechanism still needs to be verified. Moreover, the data of HBV exposure of controls selected in aforementioned GWAS study was not complete, which may introduce information bias to interpret the results.

Therefore, we sought to investigate and extend our understanding of the effect of these two recently identified SNPs in the risk of CHB in two large independent populations with different infection rate of HBV, one from Northern Chinese Han and another from Southern Chinese Zhuang, respectively in this study. Importantly, each population in this study included CHB patients and individuals spontaneously recovered to ensure all the subjects have the history of HBV exposure.

## Methods

### Study population and design

Two different populations were enrolled in this study; the first population was unrelated Han ethnicity, recruited from Beijing, Northern China and second one was unrelated Zhuang Chinese, from Southern China. Each population was composed of two subgroups: spontaneously recovered individuals with history of HBV infection and CHB patients, according to serologic tests, HBV virological index, liver function indexes and symptoms of hepatitis B. The subject was diagnosed as chronic HB when his/her serum levels of alanine aminotransferase and asparate aminotransferase were continuously abnormal, and HBsAg was seropositive and anti-HBs were seronegative after six months of acute infection. The criteria of spontaneously recovered infection were as follows: positive for both anti-HBs and anti-HBc antibodies, definitely negative for HBsAg, normal liver functional tests, and no history of acute/chronic hepatitis B and HBV vaccination.

The Han population included 1518 Han subjects, among which 736 CHB patients were from Beijing Ditan Hospital and Beijing You'an Hospital during the period of November 2001 to August 2004 and 782 spontaneously recovered subjects who were screened from ∼5000 individuals from serological investigation on HBV infection in Beijing, 2006 [22]. The Chinese Zhuang was composed of 177 CHB patients and 208 spontaneously recovered subjects, who were randomly selected from serological investigation on HBV infection in Southern China, 2006. Written informed consent was obtained from each subject. The Institutional Review Board at the Institute of Medical Sciences, Chinese Academy of Medical Sciences approved the study.

### Serological testing

Enzyme-linked immunoadsorbent assay was used to detect the serum HBsAg, anti-HBs and anti-HBc (IMX; Abbott Diagnostics, North Chicago, IL, USA).

### Polymorphism genotyping

Genomic DNA was extracted from blood sample of all subjects by using QIAamp-Blood Kit (Qiagen, Hilden, Germany). All the subjects were genotyped using TaqMan Genotyping system (Applied Biosystems, Foster City, CA) without knowledge of the subjects' infection status. PCR was performed using TaqMan Universal Master Mix on the Bio-Rad iQ™5 Real-Time PCR Detection System (Bio-Rad Laboratories, Hercules, CA) and the program was heating to 95°C for 10 minutes followed by 40 cycles of 92°C for 15 seconds and 60°C for 1 minute. Fifteen percent of the masked sample was randomly selected and genotyped twice by different people independently. The reproducibility was 100%.

### Statistical analysis

Chi-square test was also used to compare the allele and genotype frequencies of the two SNPs between CHB patients and spontaneously recovered individuals in each population. The odds ratios (ORs) and their 95% confidence interval (CI) were calculated to estimate the associations between the genotype and the risk of infection status using logistic regression model. All of above statistical analyses were performed using Statistical Analysis System software (version 9.13; SAS Institute, Cary, NC). PHASE v2.1 [23] was used to estimate the haplotype frequencies composed of two SNPs and their difference in CHB and spontaneously recovered individuals was calculated using chi-square test as well. Estimation of LD, including r^2^ and D', and haplotype association analyses were conducted using Haploview [24].

## Results

### Study population

All the subjects in this study had the history of HBV exposure. The Chinese Han population consisted of 1518 subjects (1,000 males and 518 females) and their mean age was 39.4±14.3 years. And the Chinese Zhuang was comprised of 385 individuals (235 males and 150 females) and their mean age was 40.3±11.6. No significant gender difference between the CHB and spontaneously recovered individuals in both populations. However, the spontaneously recovered individuals (mean age: 43.2±15.6) was significantly older than the CHB patients (mean age: 35.5±11.6) in Han ethic groups (*P*<0.0001), and vice versa in Zhuang ethic groups (41.2±11.6 vs. 39.4±11.5, *P* = 0.146), respectively. The details were shown in Table 1.

**Table 1:** Characteristics of the subjects from two case-control studies.

	Han Chinese				Zhuang Chinese			
	Total (%)	Cases (%)	Controls (%)	*P*	Total (%)	Cases (%)	Controls (%)	*P*
	1518	736	782		385	177	208	
Age at enrollment, years	39.4±14.3	35.5±11.6	43.2±15.6	<0.0001	40.3±11.6	41.2±11.6	39.4±11.5	0.146
Gender								
Male	1000 (65.9)	492 (66.9)	508 (65.0)	0.439	235 (61.0)	101 (57.1)	134 (64.4)	0.140
Female	518 (34.1)	244 (33.2)	274 (35.0)		150 (39.0)	76 (42.9)	74 (35.6)	

### Association between frequency of rs3077 and rs9277535 and clinical outcomes of HBV infection

The results of each SNPs association analysis are summarized in table 2. We found that A allele of rs3077 was less frequent in CHB patients than in spontaneously recovered subjects among the Han population (28.1% vs. 42.8%, *P* = 3.714×10^−17^). The same trend was shown in Chinese Zhuang (19.7% vs. 23.6%), but the latter didn't reach statistically significance (*P* = 0.206). Intriguingly, A allele of rs9277535 also showed a significantly protect effect against CHB not only in Chinese Han but also in Chinese Zhuang, with 35.8% and 44.6% in CHB and spontaneously recovered subjects in Han Chinese (*P* = 8.828×10^−7^) and 23.4% and 34.0% in Chinese Zhuang (*P* = 0.001), respectively. In addition, the frequency of A allele in spontaneously recovered subjects was significantly lower in Chinese Zhuang than in Chinese Han both for rs3077 (23.6% vs. 42.8%) and rs9277535 (33.9% vs. 44.6%), with the p values of 8.621×10^−13^ and 8.217×10^−15^, respectively, which may partially explain the different prevalence of CHB in these two population.

**Table 2:** The association between rs3077 and rs9277535 and the outcomes of HBV infection.

SNP ID	Han ethnicity				Zhuang ethnicity			
	Chronic HB (%)	Spontaneously recovered subjects (%)	OR (95% CI)	*P*	Chronic HB (%)	Spontaneously recovered subjects (%)	OR (95% CI)	*P*
rs3077								
GG	391 (53.1)	262 (33.5)			115 (65.0)	125 (60.1)		
AG	276 (37.5)	371 (47.4)	0.540 (0.464–0.628)†	4.068×10^−16^	54 (30.5)	68 (32.7)	0.811 (0.580–1.135)†	0.221
AA	69 (9.4)	149 (19.1)			8 (4.5)	15 (7.2)		
A allele	414 (28.1)	669 (42.8)	0.523 (0.450–0.609)‡	3.714×10^−17^	70 (19.7)	98 (23.6)	0.800 (0.566–1.130)‡	0.206
rs9277535								
GG	306 (41.6)	239 (30.6)			104 (58.8)	93 (44.7)		
AG	332 (45.1)	388 (49.6)	0.696 (0.601–0.806)†	1.062×10^−6^	63 (35.6)	89 (42.8)	0.606 (0.441–0.833)†	0.002
AA	98 (13.3)	155 (19.8)			10 (5.7)	26 (12.5)		
A allele	528 (35.8)	698 (44.6)	0.694 (0.600–0.803)‡	8.828×10^−7^	83 (23.4)	141 (33.9)	0.597 (0.434–0.822)‡	0.001

† Additive model was used to estimate the OR with GG genotype as the reference;

‡ G allele was used as reference.

Logistic regression model was subsequently used to determine the two SNPs' effects. Among Chinese Han groups, the subjects with at least one A allele of rs3077 had a decreased risk to CHB compared with the subjects with GG genotype (OR = 0.540, 95%CI: 0.464–0.628, *P* = 4.068×10^−16^). And the same trend was observed in rs9277535 (OR = 0.696, 95%CI: 0.601–0.806, *P* = 1.062×10^−6^). Similarly, in Chinese Zhuang groups, the subjects carrying at least one A allele of rs9277535 also protect against the CHB compared with GG carriers (OR = 0.606, 95%CI: 0.441–0.833, *P* = 0.002), but not the same with rs3077, which was not observed significantly difference between the CHB and spontaneously recovered subjects.

Since the prevalence of HBsAg is significantly higher for males (8.6%) than females (5.7%), stratified analysis was used to test the association of these two SNPs and CHB in males and females, separately, but no significant difference was observed (data not shown).

We further constructed the haplotype composing of the rs3077 and rs9277535 (data was shown in Table 3) and found that individuals carrying haplotype A-A had the lowest risk of CHB compared with G-G haplotype both in Chinese Han and Chinese Zhuang. Moreover, strong linkage disequilibrium was not detected between these two SNPs, with the D' and r^2^ equaling 0.569 and 0.265 in Chinese Han, and 0.408 and 0.113 in Chinese Zhuang, respectively.

**Table 3:** Comparison of haplotype frequencies between CHB patients and controls.

	Han ethnicity				Zhuang ethnicity			
Haplotype (rs3077–rs9277535)	Chronic HB (%)	Spontaneously recovered subjects (%)	OR (95% CI)	*P*	Chronic HB (%)	Spontaneously recovered subjects (%)	OR (95% CI)	*P*
GG	848 (57.6)	680 (43.5)	1.00		235 (66.4)	246 (57.7)	1.00	
GA	210 (14.3)	215 (13.8)	0.783 (0.632–0.971)	0.026	49 (13.8)	72 (18.8)	0.642 (0.430–0.957)	0.030
AG	96 (6.5)	186 (12.0)	0.414 (0.317–0.540)	3.430×10^−11^	36 (10.2)	29 (8.4)	1.050 (0.638–1.730)	0.847
AA	318 (21.6)	483 (30.9)	0.528 (0.444–0.628)	4.400×10^−13^	34 (9.6)	69 (15.1)	0.551 (0.350–0.868)	0.010

## Discussion

The nature history of HBV infection is complicated and it is necessary to find clinical and genetic markers to aid in prediction of those who will have a higher risk to develop CHB and worse outcomes, such as liver cirrhosis and HCC. Accumulating evidence indicated that individuals who will clear HBV infection have a low risk of viral persistence [25]. Clearance of HBV infection is associated with vigorous CD4^+^ T cells [26] and HLA class II glycoprotein presenting viral peptide to CD4^+^ T cells and considered to be critical for the immune response [4], [17], [18], [26]–[28], indicating genetic polymorphisms in HLA system may be implicated in the clinical outcomes of HBV infection.

Nevertheless, the effect of HLA genes on the HBV persistent infection remains contradictory. The recent GWAS study has identified two SNPs located at HLA-DP are associated with CHB in Japanese [21]. HLA loci are among the most polymorphic in the human genome and even the same HLA loci, the frequency of allele may have much difference among diverse populations. So it is important to validate the effects of above SNPs among different population which may deepen our understanding of the genetic susceptibility of CHB in different population.

Identification of genetic risk factors of CHB in Chinese is especially crucial since China is the one of the highest prevalence area in the world. Intriguingly, even in China, the rate of CHB is also diverse among different regions and different ethnic populations. Epidemiological studies reported that the prevalence rate of HBsAg positive of Chinese Han in Beijing is relative low (3.49%) [29], while Chinese Zhuang had the highest HBsAg prevalence (13.4%) [3]. We therefore selected these two ethnic populations to evaluate the association between these two SNPs and the clinical outcomes of HBV exposure. We found the protection effect of the A allele of both rs3077 and rs9277535 in the risk of CHB in Chinese Han, which is consistent with the results from Japanese GWAS study. However, only rs9277535 was shown to be significantly associated with reduced risk of CHB in Chinese Zhuang. The reason that the positive findings in Japanese can be completely and partly repeated in our Northern Chinese Han and Southern Chinese Zhuang respectively may be explained by the share considerable proportion of genetic components with Japanese [30]. According to a recent genome-wide study of genetic diversity of Asian populations [30], Northern Chinese Han is the one closely related to Japanese except Korean on the phylogenetic tree and Zhuang is more southern compared with Japanese which is typical representative of northern population. And another reason for the non-significantly protection effect of rs3077 in Chinese Zhuang may be due to the small sample for Chinese Zhuang in this study. Since the sample from Chinese Zhuang only included 177 CHB and 208 spontaneously recovered subjects, it had only 14.4% power to detect an OR of 0.8 under the A allele frequency of 0.236.

It has been well documented that the prevalence of HBV infection is much higher in Asian than in Europeans. Conceivably, the diverse frequency analysis in HapMap dataset demonstrated that the protective A allele of rs9277535 was significantly higher in European population (0.739) than in Beijing Han population (0.463). Moreover, we found that the frequencies of both A allele of rs3077 and rs9277535 in Chinese Han were similar with those reported in Beijing Han of HapMap, while much higher than the counterparts of Chinese Zhuang in Guangxi. These results were consistent with the epidemiological data, indicating the higher infection rate of HBV in Zhuang may partially due to lower frequencies of these two SNPs. It is worth mentioning that just when this manuscript was preparing, Guo and colleagues reported genetic variants in the HLA-DP locus were strongly associated with persistent HBV infection in the Han Chinese population, which was consistent with our finding [31].

The rs3077 and rs9277535 locate at the 3′-untranslated regions (UTR) of HLA DPA1 and DPB1, respectively, which belong to the third set of classical HLA class II loci coding for the DP alpha and beta chains and may play substantial roles in antigen presentation to CD4^+^ positive T lymphocyte. Studies have shown that HLA-DPs are highly polymorphic, with 28 DPA1 and 138 DPB1 alleles till now (http://www.ebi.ac.uk/imgt/hal/). Functional analysis of HLA-DP polymorphisms in the coding region suggested that DPβ residues 9, 11, 35, 55, 56, 69 and 84–87 play a key role in T cell allorecognition and peptide binding [32] and the DPA1 allele difference has been observed to significantly alter T cell recognition [33]. Also, a single K to E difference at position 69 of DPB1 was found to influence T cell recognition [32], [34]. All above evidence supports that the genetic polymorphisms in HLA DP region may result in the variable capacity of antigen presentation and influence the outcomes of Hepatitis B infection finally.

In terms of function of rs3077 and rs9277535, the clear mechanism is still unknown. Both rs3077 and rs9277535 locate at the 3′-UTR of HLA-DPA1 and DPB1 gene, respectively, their SNPs are impossible to affect the antigen-binding site directly. But they may be the binding site of some microRNA (miRNA), a group of short non-coding RNAs involved in post-transcriptional regulation of gene expression by base paring with target mRNAs at the 3′-UTR, leading to affect both the stability and translation of mRNA. SNPs located at miRNA-binding site are likely to disrupt miRNA-target interaction, and result in the deregulation of target gene expression [35]. Alternatively, these two SNPs may have strong LD with other SNPs which can influence the capacity of antigen presentation in the coding region since they have long LD with other SNPs in HLA-DP region in Chinese Han according to HapMap data (data not shown), acting as a marker of other potentially functional loci. Nonetheless, further functional analysis of HLA-DP region, including these two SNPs, still needs to be clarified.

Our study has several strengths. Firstly, we use the two independently populations with different HBV infection rate to do the replication and validation, which rigorously strength the plausibility of this study [36], [37]. Secondly, spontaneously recovered individuals but not general population was selected as controls to ensure all the subjects in the study having the history of HBV exposure, reducing the selection bias of study. Moreover, all the spontaneously recovered individuals were population based but not hospital based enrolled. They were screened from serological investigation on HBV infection among general population in Beijing or Southern China in 2006 and showed good representativeness. Thirdly, we can ensure our genotyping is accurate since the allele frequency of these two SNPs in our Han Chinese is comparable to those reported in Beijing Han of HapMap data, with similar LD of these two SNPs in our study (D' = 0.569, r^2^ = 0.265) with those from HapMap (D' = 0.574, r^2^ = 0.245). Notwithstanding, the case-control population from Chinese Zhuang was not statistically enough, which may lead to the false-negative results of rs3077.

In conclusion, our study support that rs3077 and rs9277535 is significantly related to HBV persistent infection and both A alleles of these two SNPs are protection alleles, which are in accord with the previous results from Japanese. These findings might provide further evidence for the importance of the HLA's role in HBV infection and provide potential implications for therapeutic guidance. However, further function analyses were warranted to validate the biological plausibility of these two SNPs in hepatitis B infection.
